# Epicutaneous immunotherapy for food allergy: A systematic review and meta‐analysis

**DOI:** 10.1002/clt2.70045

**Published:** 2025-03-02

**Authors:** Péter Csonka, Bohee Lee, Ilari Kuitunen

**Affiliations:** ^1^ Faculty of Medicine and Health Technology Tampere Center for Child Adolescent and Maternal Health Research Tampere University Tampere Finland; ^2^ Terveystalo Healthcare Tampere Finland; ^3^ National Heart and Lung Institute Imperial College London London UK; ^4^ Kuopio Pediatric Research Unit (KUPRU) University of Eastern Finland Institute of Clinical Medicine Kuopio Finland; ^5^ Department of Pediatrics Kuopio University Hospital Kuopio Finland

**Keywords:** allergen immunotherapy, cow's milk, epicutaneous immunotherapy, EPIT, peanuts

## Abstract

**Background:**

Food allergies pose a global healthcare challenge, underscoring the need for effective interventions. This study evaluated the efficacy and safety of epicutaneous immunotherapy (EPIT) for food allergen desensitisation.

**Methods:**

We conducted a systematic review of randomised controlled trials by searching Ovid EMBASE, PubMed and Scopus in April 2024. Using a random‐effects meta‐analysis, we evaluated the clinical effectiveness and harms of EPIT, reporting results as risk ratios with 95% confidence intervals (CI).

**Results:**

After screening 460 abstracts and 35 full reports, 11 were included: nine on peanuts and two on cow's milk (CM). Peanut EPIT had a 51.2% treatment response versus 22.4% for placebo (RR 2.16, CI 1.49–3.12; four studies; moderate certainty). The RR for milk EPIT response rate was 1.78 (CI 1.06–3.00; one study). Five peanut studies (1396 patients) reported EPIT‐related adverse events (RR 1.39, CI 0.94–2.05; low certainty).

**Conclusions:**

EPIT offers a moderate treatment response with a favourable safety profile and significant improvements in quality of life. Current knowledge of EPIT remains limited, with evidence confined to peanut and CM allergies. There is a lack of research on sustained unresponsiveness achieved through food EPIT.

## 1 INTRODUCTION

Food allergies represent a significant public health concern with the potential to cause life‐threatening reactions. While some food allergies, such as those to cow's milk (CM) and hen's eggs, may be outgrown by many children, peanut allergy is typically lifelong, with resolution occurring in fewer than 25% of cases.^1^ Although the safety of food allergen immunotherapy (AIT) remains a subject of ongoing debate,^2^ it has emerged as a viable option for increasing tolerance and reducing the risk of severe allergic reactions, offering a potential avenue for managing persistent allergies.^3^

Immunotherapy for food allergies is mainly administered through sublingual immunotherapy (SLIT), oral immunotherapy (OIT), and epicutaneous (epicutaneous immunotherapy (EPIT)) routes, while subcutaneous immunotherapy is generally considered unsafe for food allergies.^3^ AIT aims to increase the allergen threshold required to elicit a reaction, thereby reducing the risk of adverse events due to accidental exposure. Allergen‐specific immunotherapy works by inducing regulatory T (Treg) cells to produce immunosuppressive cytokines such as IL‐10, TGF‐β, and IL‐35 and by enhancing the expression of inhibitory molecules such as cytotoxic T‐lymphocyte antigen 4 and programed cell death protein 1. This results in the suppression of T helper 2 (Th2) cells, basophils, and eosinophils and the promotion of allergen‐specific regulatory B (Breg) cells. Collectively, these mechanisms contribute to a reduced production of allergen‐specific immunoglobulin E (sIgE) and an increase in allergen‐specific IgG_4_ (specific IgG4 (sIgG4)), fostering allergen‐specific tolerance.^4^

In AIT trials, two key outcomes are prioritised for their clinical relevance and importance to patients and caregivers: desensitisation and sustained unresponsiveness (SU).^5^ Desensitisation refers to the ability to tolerate a specific dose of the allergen while on therapy, whereas SU describes continued tolerance even after therapy cessation. Ideally, these outcomes should be evaluated using double‐blind, placebo‐controlled food challenges (DBPCFC). However, the lack of a standardised protocol across studies makes it challenging to compare trial results.^5^, ^6^, ^7^

Epicutaneous immunotherapy is a relatively novel non‐invasive method that involves the application of allergen‐containing patches on intact skin.^3^, ^8^ This approach takes advantage of the skin's immune properties, particularly the activation of Langerhans cells and specific dendritic cells, to induce Treg cells and downregulate the Th2 response.^9^, ^10^ EPIT's localised allergen exposure minimises systemic allergic reactions, potentially providing a safer alternative to other immunotherapy methods.^11^ This is the first systematic review to comprehensively assess all published clinical trials on food EPIT. It covers each studied food allergen and provides an in‐depth evaluation of efficacy, safety, adverse events, relevant biomarkers, and a formal quality assessment.

## 2 MATERIALS

We reported this review according to the Preferred Reporting Items for Systematic Reviews and Meta‐Analyses guidelines, with the checklist provided in the Supporting Information S1.^12^ The protocol for this review was registered to PROSPERO 2024 CRD42024549559 and is available from https://www.crd.york.ac.uk/prospero/display_record.php?ID=CRD42024549559.

### 2.1 Study design and search process

We conducted a systematic review by searching the Ovid EMBASE, PubMed and Scopus databases on 30^th^ April 2024. The complete search strategy is provided in the Supporting Information S1. Grey literature was not included. Search results were uploaded to Covidence (Veritas Healthcare Inc, Victoria, Australia) software for title and abstract screening and full text screening. Two authors (BL and IK) independently screened title/abstracts and full texts. Disagreements were resolved by consensus or by consulting the third author (PC). During the screening, we employed Covidence's automatic RCT recognition tool to exclude non‐RCT studies. This tool has demonstrated high accuracy and validity for use in systematic reviews.^13^

### 2.2 Inclusion and exclusion criteria

We included studies focussing on patients (children and adults) with IgE‐mediated allergic disease deemed eligible for desensitisation therapy. The intervention needed to be EPIT, with the control condition being placebo, an alternative desensitisation therapy, or no intervention. Outcomes were not specified to include a wide range of outcomes. Only parallel‐group randomised controlled trials were included. Reports not written in English and observational studies were excluded. We excluded conference abstracts which often present preliminary results, to ensure the quality of the studies assessed as no established guidelines exist for their evaluation.

### 2.3 Data extractions

One author conducted the data extraction, and a second author validated the extracted data by checking the extracted data for accuracy. From each included study, we extracted the following information: study period, country, intervention, control, patient characteristics (e.g. age, allergic comorbidities, baseline laboratory exam results), primary and secondary outcomes, effect estimates, and funding details.

### 2.4 Main objectives

Our primary objective was to assess the proportion of patients who achieved a substantial increase in allergen consumption. We classified treatment response in line with the original studies, where a significant response was defined as a 10‐fold increase in tolerance across the individual studies. Secondary objectives included assessing SU, QoL, serum peanut and CM‐specific IgE‐ and IgG_4_‐levels, intervention tolerance, and adverse events.

### 2.5 Risk of bias

The risk of bias was evaluated using the Cochrane Risk of Bias 2.0 tool.^14^ Assessments were conducted separately for objective and subjective outcomes (Table S1). Two authors independently performed the risk of bias evaluation and reached consensus on the final assessment.

### 2.6 Synthesis

Outcomes were pooled when at least two studies reported comparable outcomes. Dichotomous outcomes were analysed using a random‐effects inverse variance meta‐analysis (DerSimonian and Laird), and risk ratios with 95% confidence intervals (CI) were calculated for both the clinical effectiveness and treatment emergent adverse events. Publication bias was analysed when at least five studies analysed the same outcome by visually examining funnel plots and performing trim‐and‐fill analysis. Statistical heterogeneity was analysed using Cochran's Q, and inconsistency was interpreted based on the I^2^‐value; however, this did not guide the random‐effects model choice and was mostly used in the evidence certainty assessment. Other outcomes from the included studies were systematically summarised following the synthesis without meta‐analysis (synthesis without meta‐analysis) guidance.^15^

Evidence certainties for the main outcomes of clinical effectiveness and total treatment‐emergency adverse events were assessed using the Grading of Recommendations, Assessment, Development, and Evaluations framework.^16^ Evidence certainties were not assessed for outcomes for which pooled analysis was not performed.

## 3 RESULTS

### 3.1 Search results

**FIGURE 1:**
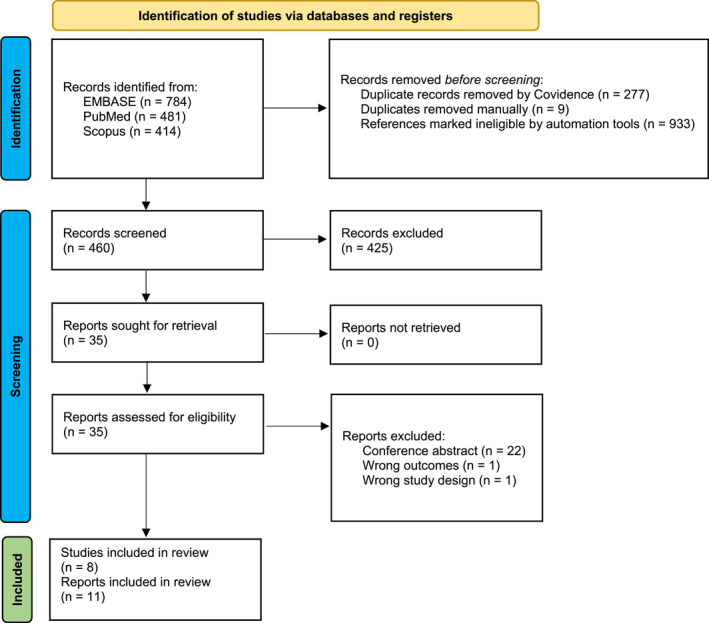
Preferred Reporting Items for Systematic Reviews and Meta‐Analyses (PRISMA) flowchart of the study selection process.

The initial searches retrieved 1679 results. After removing duplicates and references that were marked as non‐RCTs by automation tools, a total of 460 abstracts were assessed. Finally, 35 reports were assessed, and eight studies with 11 reports were included for analysis (Figure 1).

### 3.2 Study and participant characteristics

Of the reports included, nine focussed on peanuts and two on CM. The nine peanut‐related studies were from six distinct trials, encompassing a broad age range of 1–55 years (Table 1). In the peanut trials, the most commonly administered dose was 250 μg, but doses of 100 and 50 μg were also used (Table 1). The primary peanut studies were double‐blinded, some with open‐label extension phases to allow for prolonged assessment. Due to differing inclusion criteria, the mean and median ages in the peanut studies varied considerably; however, age distributions were comparable across study groups (Table 2). Peanut sIgE levels did not significantly differ between the intervention and placebo groups.

**TABLE 1:** Characteristics of the included studies.

Study	Trial	Other	Country	Study period	Intervention	Comparator	Age group	Blinding	Funding	Conflict of interest
Peanut										
Davis, 2023^17^	PEPITES and REALISE	Safety in atopic patients								
DunnGalvin, 2021^18^	PEPITES and PEOPLE	Quality of life extension								
Fleischer, 2019^19^	PEPITES	Original work	Australia, Canada, Germany, Ireland, the USA	2016–2018	250 μg peanut patch	Placebo	4–11 years	Double	Industrial funding	Reported
Fleischer, 2020^20^	PEOPLE	Open‐label extension to PEPITES	Australia, Canada, Germany, Ireland, the USA	2016–2020	250 μg peanut patch		4–11 years	Open‐label extension	Industrial funding	Reported
Greenhawt, 2023^21^	EPITOPE	Original work	Australia, Canada Europe, and the USA	2017–2022	250 μg peanut patch	Placebo	1–3 years	Double	Industrial fundings	Reported
Jones, 2017^22^	Consortium for food allergy research	Original work	The USA	Not reported	100 μg and 250 μg peanut patch	Placebo	4–25 years	Double	Industrial funding	Reported
Pongracic, 2022^23^	REALISE	Original work	Canada and the USA	2016–2017	250 μg peanut patch	Placebo	4–11 years	Double and open‐label extension	Industrial funding	Reported
Sampson, 2017^24^	‐	Original work	Canda, Europe, and the USA	2012–2016	50 μg, 100 μg, and 250 μg peanut patch	Placebo	6–55 years	Double	Industrial funding	Reported
Scurlock, 2021^25^	Consortium for food allergy research	Quality of life extension								
Milk										
Dupont, 2010^26^	‐	Original work	France	Not reported	1 mg milk patch	Placebo	3 months to 15 years	Double	Industrial funding	Reported
Petroni, 2024^27^	‐	Original work	Canada and the USA	2014–2017	150–500 μg milk patch	Placebo	2–17 years	Double	Industrial funding	Reported

**TABLE 2:** Characteristics of the included patients.

		Number of participants (n)		Age (years)		Peanut/Cow's milk sIgE at baseline (kU/L)		Comorbidities	
Study	Trial	Intervention	Control	Intervention	Control	Intervention	Control	Intervention	Control
Peanut									
Fleischer, 2019^19^	PEPITES	238	118	Median 7 (IQR 6–9)	Median 7 (IQR 5–9)	Median 78 (IQR 1–1008)	Median 101 (IQR 1–1104)	Asthma 49%	Asthma 44%
								AD 58%	AD 67%
								Rhinitis 56%	Rhinitis 57%
								Other allergy 86%	Other allergy 85%
Greenhawt, 2023^21^	EPITOPE	244	118	Median 3 (IQR 2–3)	Median 2 (IQR 1–3)	Median 13 (IQR 4–66)	Median 15 (IQR 5–52)	Asthma 16%	Asthma 23%
								AD 80%	AD 81%
								Rhinitis 20%	Rhinitis 20%
								Other allergy 66%	Other allergy 69%
Jones, 2017^22^	Consortium for food allergy research	49 (24, 100 μg; 25, 250 μg)	25	Median 8 (IQR 4–17)	Median 9 (IQR 5–20)	Median 92 (IQR 1–202)	Median 58 (IQR 1–213)	Asthma 59%	Asthma 48%
								AD 53%	AD 48%
								Other allergy 84%	Other allergy 80%
Pongracic, 2022^23^	REALISE	164	65	Mean 7.2 (SD 2.2)	Mean 7.2 (SD 2.3)	Median 91 (IQR 48–274)	Median 90 (IQR 51–298)	Asthma 46%	Asthma 47%
								AD 59%	AD 54%
								Rhinitis 60%	Rhinitis 56%
								Other allergy 85%	Other allergy 83%
Sampson, 2017^24^		165 (53; 50 μg; 56, 100 μg; 56, 250 μg)	56	Median 11 (IQR 8–16)	Median 11 (IQR 9–14)	Median 80 (IQR 32–213)	Median 69 (IQR 17–212)	Not reported	Not reported
Cow's milk									
Dupont, 2010^26^	‐	10	8	Mean 4 (SD 2)		Mean 20 (SD 23)	Mean 12 (SD 17)	Not reported	Not reported
Petroni, 2024^27^	‐	145	53	Mean 8 (SD 4)	Mean 8 (SD 4)	Median 64 (IQR 32–159)	Median 61 (IQR 25–163)	Asthma 70%	Asthma 71%
								AD 71%	AD 68%
								Rhinitis 74%	Rhinitis 70%
								Other allergy 90%	Other allergy 89%

Abbreviation: SD, standard deviation.

Both CM studies employed Viaskin patches and were double blind. The milk doses ranged from 150 to 1000 μg (Table 1) and involved paediatric participants with mean ages of 3.8 and 8.0 years (Table 2). Both studies reported CM sIgE levels, finding no significant differences between the active and placebo groups. Only one study reported CM sIgG4 levels.

The prevalence of atopic comorbidities was similar between the intervention and control groups in both the peanut and CM studies. Most patients in these EPIT studies had additional food allergies. All studies included were funded by industry sponsors (Table 2).

### 3.3 Risk of bias

**FIGURE 2:**
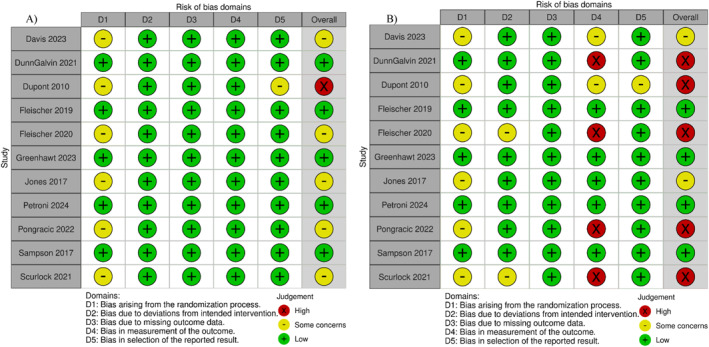
Risk of bias assessments according to the RoB 2.0 tool to (A) objective and (B) subjective outcomes.

The overall risk of bias for objective outcomes was low in five studies, raised some concerns in five studies, and was high in one study (Figure 2A). The primary source of bias in these cases stemmed from the randomisation process. For subjective outcomes, the overall risk of bias was low in four studies, raised some concerns in two studies, and was high in five studies (Figure 2B). The main sources of bias were the randomisation process and outcome measurement, as the studies with a high risk of bias were open label.

### 3.4 Clinical effectiveness of peanut epicutaneous immunotherapy

**FIGURE 3:**
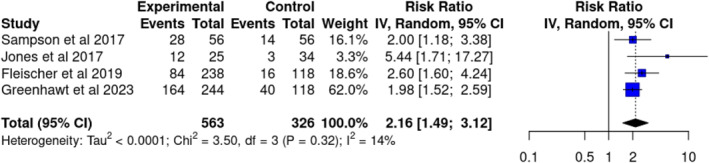
Forest plot of the clinical effectiveness of peanut epicutaneous immunotherapy (EPIT) treatment. Effectiveness calculated as treatment responder and treatment response meaning at least ten‐fold increased in the tolerated peanut dose.

Four original trials measured the treatment response rate to EPIT 250 μg after 12 months, involving a total of 889 patients. The treatment response rate was 51.2% in the EPIT group and 22.4% in the placebo group, with an RR of 2.16 (CI 1.49–3.12; Figure 3). The certainty of the evidence was rated as moderate and was downgraded once due to risk of bias. The number needed to treat was 3.5 (CI 2.1–5.6), corresponding to an absolute improvement of 29 per 100 individuals. Two studies also used an EPIT dose of 100 μg, while one study additionally used a dose of 50 μg; all showed improvement compared to placebo (Table 3).

**TABLE 3:** Effectiveness outcomes in peanut and cow's milk (CM) epicutaneous immunotherapy (EPIT) trials.

Study (trial)	Definition or response/success	Treatment response/success			Timing (week)
		Intervention	Control	Estimate	
Peanut					
Fleischer, 2019^19^ (PEPITES)	With baseline ED <10 mg if post treatment ED >300 mg; with baseline ED 10–300 mg if post treatment ED >1000 mg.	250 μg 84/238 (35%)	16/118 (14%)	RR 2.6 (CI 1.6–4.2)	52
Greenhawt, 2023^21^ (EPITOPE)	With baseline ED ≤10 mg if post treatment ED ≥300 mg; with baseline ED >10 mg if post treatment ED >1000 mg	250 μg 164/244 (67%)	40/118 (34%)	RR 2.0 (CI 1.5–2.6)	52
Jones, 2017^22^ (Consortium for food allergy research)	Passing a 5044 mg protein OFC or achieving a 10‐fold increase in ED over baseline	250 μg 12/25 (48%)	3/24 (12%)	RR 3.8 (1.2–12.0)	52
		100 μg 11/24 (46%)		RR 4.0 (1.3–12.5)
Sampson, 2017^24^	ED ≥ 1000 mg	250 μg 28/56 (50%)	14/56 (25%)	RR 2.0 (CI 1.2–3.4)	52
		100 μg 23/56 (41%)		RR 1.6 (CI 0.9–2.8)	
		50 μg 24/53 (45%)		RR 1.8 (CI 1.0–3.1)	
Sampson, 2017^24^	≥10‐times increase in ED	250 μg 23/56 (41%)	10/56 (17.9)		52
		100 μg 14/56 (25%)			
		50 μg 16/53 (30%)			
Cow's milk					
Dupont, 2010^26^	Cumulative tolerated dose	Increase of 22 mL in the EPIT group, compared to and 1.1 mL in the placebo group (*p* = 0.13).	Not specified	Not specified	12
Petroni, 2024^27^	10‐Fold increase in cumulative relative dose	Statistical significance response in the 2‐11‐year‐old subgroup 22/38 (57.9%) in the 300 μg EPIT group	13/40 (32.5%)	RR 1.78 (CI 1.06–3.00)	52

Abbreviations: ED, eliciting dose; OFC, oral food challenge.

The original studies also included open‐label extensions and published secondary analyses that further examined effectiveness.^20^, ^25^ In the 2‐year follow‐up of the PEOPLE study, which extended the findings of PEPITES, the treatment response rate remained consistently high at the 36‐month assessment. Additionally, crossover from the placebo group also enhanced the treatment response rate. Similar findings were observed in the Consortium of Food Allergy Research study, where all groups demonstrated improved treatment response rates at the 36‐month mark. A combined analysis of data from PEPITES and REALISE further performed the treatment response in subgroups of patients with atopic dermatitis, asthma, and other food allergies.^17^ The results indicated that patients with atopic comorbidities had higher treatment response rates than those without. None of the peanut studies evaluated SU.

Serum peanut sIgE and sIgG4 levels were measured in five studies, all of which reported similar patterns for both laboratory values. sIgE levels initially increased during the first 6 months and then returned to baseline or lower levels when follow‐up extended beyond 12 months (Table 4). In contrast, sIgE levels remained stable in the placebo groups. Peanut sIgG4 levels increased progressively over the 12‐month period with peanut patches. Only minor or no fluctuations in peanut sIgG4 were observed within the placebo groups (Table 4).

**TABLE 4:** Laboratory measurements.

Study (Trial)	Change in S‐IgE	Change in S‐IgG_4_
Peanut		
Fleischer, 2020^20^ (PEOPLE)	IgE levels increased until 6 months and turned to decrease then and were below the baseline from 18 to 36 months in all EPIT patients who responded to treatment. In non‐responders the IgE levels remained higher than baseline throughout the 36 months.	In the per protocol data set the median levels of peanut sIgG_4_ increased significantly in the active group.
Greenhawt, 2023^21^ (EPITOPE)	IgE levels decreased from baseline to 12 months in the EPIT group and continued to increase in the placebo group.	Peanut sIgG_4_ level increased from baseline to month 12 in the intervention group, with little change in the placebo group.
Jones, 2017^22^ (Consortium for food allergy research)	Both EPIT groups showed increase in levels at three and 6 months and decrease towards baseline at 1 year. Placebo group had more stable IgE‐level throughout the 12‐month follow‐up.	Increases in peanut sIgG_4_ levels and IgG_4_/IgE ratios were observed in peanut EPIT‐treated participants.
Sampson, 2017^24^	All EPIT groups showed an increase in levels in 3‐ and 6‐month timepoint, but the IgE levels returned to baseline at 12 months. Placebo group had stable IgE level.	Peanut sIgG_4_ levels increased progressively over the 12‐month period with peanut patches. Only minor fluctuations were observed with the placebo patch.
Scurlock, 2021^25^ (Consortium for food allergy research)	The IgE levels in all groups decreased below the baseline during the 12–36 months of follow‐up.	Peanut and Arah2 sIgG_4_ increases were observed in all treatment groups over time, with the largest increase noted in the VP250 group.
Cow's milk		
Dupont, 2010^26^	EPIT did not increase CM protein–sIgE in either the active or placebo group.	Did not report CM sIgG_4_ values.
Petroni, 2024^27^	Slight transitional increase in CM sIgE in the Viaskin milk 300‐μg group only from baseline to month 3, followed by a decrease below the month 0 value at month 12. No significant trends to the component proteins.	Specific IgG4 to casein, alpha‐lactalbumin, and beta‐lactoglobulin significantly increased up to month 12 in the Viaskin milk 150, 300, and 500 μg groups, and all were significantly higher than placebo.

*Note*: Change in serum peanut and cow's milk (CM) specific IgE and IgG_4_ levels from baseline to end of trial.

### 3.5 Clinical effectiveness of cow's milk epicutaneous immunotherapy

Petroni et al. evaluated the clinical effectiveness of EPIT treatment at 12 months by performing DBPCFC and also examining laboratory values.^27^ Treatment response was defined as a 10‐fold increase in cumulative relative dose (Table 3). The study found that the highest treatment response rate was seen in the 300 μg EPIT group, achieving statistical significance in the 2‐ to 11‐year‐old subgroup. In this group, the treatment response rate was 57.9% (22 of 38) in the 300 μg EPIT group compared to 32.5% (13 of 40) in the placebo group, with an RR of 1.78 (CI 1.06–3.00). Changes in casein, alpha‐lactalbumin, and beta‐lactoglobulin sIgE levels remained within CI across all groups over the 12‐month study period. Specific IgG_4_ to casein, alpha‐lactalbumin, and beta‐lactoglobulin significantly increased up to month 12 in the Viaskin Milk 150, 300, and 500 μg groups, and all were significantly higher than placebo (Table 4).

In a pilot study, Dupont et al. assessed the increase in cumulative tolerated dose at 90 days and found an increase of 22 mL in the EPIT group compared to 1.1 mL in the placebo group (Table 3). However, this difference did not reach statistical significance due to the small sample size (*p* = 0.13).^26^ The authors did not specify the oral food challenge (OFC) method used, including whether it was conducted as a DBPCFC or as an open‐label test. No notable changes were observed in CM sIgE levels after 90 days of EPIT treatment, with the mean CM sIgE remaining practically the same (20.2 vs. 19.5). This study did not report CM sIgG4 values. None of the CM studies evaluated SU.

### 3.6 Quality of life

QoL was assessed in the PEPITES study and its follow‐up, the PEOPLE study. The results indicate that QoL, as measured by food allergy QoL questionnaires, improved significantly more in the intervention group than in the placebo group during the first 12 months. By 24 months, both groups had received EPIT for either 12 or 24 months and reported improvements, as noted by both parents and children. However, the improvements appeared to be greater in the group that used the EPIT for the entire 24‐month study period. Although Petroni et al. collected QoL data in their CM EPIT study, these results have not yet been reported.

### 3.7 Harms and adverse events

A total of five studies reported on EPIT‐related adverse events for peanut trials, encompassing 1396 patients in total, with a calculated RR of 1.39 (95% CI, 0.94–2.05; Figure S1). The certainty of evidence was rated as low, with downgrades applied once for risk of bias and once for inconsistency. We did not find evidence of publication bias (Figure S2). When adverse events were categorised by severity, the risk ratios showed an upward trend correlating with increased severity. Specifically, the risk ratios were 1.15 for mild events (4 studies, 95% CI, 0.88–1.51; Figure S3), 1.71 for moderate events (4 studies, 95% CI, 1.16–2.52; Figure S4), 2.28 for severe events (5 studies, 95% CI, 1.47–3.54; Figure S5), and 1.79 for serious events (3 studies, 95% CI, 0.36–8.92; Figure S6). The risk for anaphylaxis was notably higher in the peanut EPIT group, with an absolute risk of 2.4% compared to 0.3% in the placebo group, RR 4.33 (CI 3.18–5.88; Figure S7). In the CM studies, the rates of treatment‐related adverse events were higher in the intervention groups. However, for organ‐specific adverse events, the results were highly imprecise, limiting further conclusions (Figures S8–S12).

## 4 DISCUSSION

This systematic review is the first to provide a comprehensive assessment of clinical efficacy, safety, adverse events, and immunological changes associated with EPIT for peanut and CM allergies. Our findings suggest that EPIT could serve as a viable, safe, and non‐invasive alternative to food AIT, particularly suited for paediatric populations.

The recently published systematic review on food AIT primarily focussed on efficacy outcomes reported in individual studies, rather than evaluating the overall safety and efficacy of AIT as an intervention.^5^ As a result, outcomes were pooled regardless of the allergen administration route (EPIT, OIT, or SLIT), and no formal quality assessment was conducted. Our review also extends to additional publications not included in the previous review.^17^, ^21^, ^23^, ^25^, ^26^ Concentrating exclusively on EPIT studies for food allergies allows for a more accurate comparison of treatment effects and reactivity thresholds across trials. Nevertheless, our review highlights the limited research conducted on EPIT for food allergies: we identified only nine studies on peanuts and two on CM, with no studies on other food allergens. This lack of diverse EPIT studies underscores a significant gap in the literature and an urgent need for research extending EPIT applications to other allergens.

Among the included studies, variation in OFC methodologies—specifically the lack of standardisation in dose levels and whether the OFC was conducted as double‐blind placebo‐controlled (DBPCFC) or open‐label—complicates the direct comparison of outcomes. The most commonly reported primary outcome was desensitisation, generally defined as the ability to tolerate a particular maintenance dose or a specific dose during a food challenge. While desensitisation is a critical goal, none of the studies evaluated SU, an equally important endpoint that reflects long‐term tolerance after therapy cessation. The absence of SU data limits insights into the durability of EPIT's effects and highlights another area for improvement in future EPIT trials.

The efficacy data across peanut and CM trials showed moderate success in inducing treatment response rates, with meaningful changes in allergen‐specific immunoglobulin responses. The results align with prior reports showing increases in allergen‐specific IgG4 and transient increases in allergen‐specific IgE during the initial AIT phase, followed by a decrease.^28^, ^29^, ^30^ These results may indicate a shift in the immune response towards tolerance. Initial increases in sIgE may represent a sensitisation effect, but the subsequent decrease suggests that EPIT exerts a longer‐term modulatory impact on the immune system. Peanut sIgG4 levels consistently increased across studies, further corroborating the immunomodulatory effect of EPIT in enhancing tolerance and reducing sensitivity to allergens. Similar trends were observed in CM EPIT, with statistically significant results seen primarily in younger patients and at higher doses, suggesting that age and dosage play key roles in achieving clinical efficacy.^27^

The QoL improvements observed in the PEPITES and PEOPLE studies extend EPIT's value beyond clinical desensitisation.^18^, ^19^, ^20^ Enhanced confidence in food safety, as noted by patients and caregivers, is especially relevant in paediatric settings, where food allergies significantly affect social, psychological, and educational experiences. Although limited QoL data were available for CM EPIT, the trends observed in peanut EPIT studies suggest that similar benefits may be applicable across various food allergens.

Our review found that adverse events with EPIT were common, predominantly mild to moderate in severity, and generally manageable, underscoring EPIT's favourable safety profile compared with OIT. However, studies on peanut EPIT indicated a higher absolute risk of severe adverse events and anaphylaxis though incidence rates remained low. Reporting on anaphylaxis was somewhat limited, providing only the number of cases. To better assess the risk of anaphylaxis related to EPIT, more detailed information on the timing of these reactions in relation to treatment initiation or patch changes would be valuable.

Stratified risk ratios by severity showed an increase in adverse event frequency with severity, highlighting the need for careful patient selection and monitoring, particularly for those with comorbid asthma or atopic dermatitis.^17^ Studies on CM EPIT reflected similar findings though imprecise results restricted specific conclusions about adverse event types. Moving forward, monitoring adverse events in real‐world studies will be important to understand the true incidence and profile of treatment‐emergency adverse events, especially in the long term.

While this review offers valuable insights, it also has certain limitations. Variability in study designs, participant age ranges, and dose levels complicate direct comparisons across studies. Additionally, the lack of standardised endpoints and limited long‐term follow‐up data restrict our ability to assess the sustainability of induced tolerance. Notably, no studies have examined the effects following the discontinuation of EPIT treatment. As all the studies were industry‐sponsored, we were unable to conduct any sensitivity analyses where independently funded studies would have been analysed separately. Industry‐funded studies have been found to report more positive findings and larger effect sizes. However, we have addressed this by presenting that all included studies were funded and rigorously assessed the risk of bias for each study.^31^ Another limitation is the overall risk of bias within the included studies, as only a few trials demonstrated a consistently low risk of bias.

To advance EPIT research, future studies should prioritise standardised protocols for dosing, duration, and outcome measures. Inclusion of patient‐reported outcome measures in all future AIT studies would provide valuable patient‐centred insights into the broader impacts of treatment. Long‐term studies evaluating the durability of EPIT‐induced tolerance, as well as QoL outcomes for additional allergens, would enrich our understanding of EPIT's clinical impact. Additionally, research exploring the immunological mechanisms of EPIT, particularly the roles of regulatory T and B cells and cytokine profiles, could provide key insights for optimising immunotherapy strategies for food allergies.

## 5 CONCLUSION

Epicutaneous immunotherapy shows promise as an effective and relatively safe desensitisation strategy for children with peanut and CM allergies, with notable benefits in immunological response, clinical desensitisation, and QoL. However, the increased risk of severe adverse events, particularly for peanut EPIT, warrants careful patient selection and monitoring. As EPIT becomes more widely adopted in clinical practice, continued research to refine protocols, address safety considerations, and evaluate long‐term outcomes will be essential to maximising its therapeutic potential.

## AUTHOR CONTRIBUTIONS

**Péter Csonka**: Conceptualization; data curation; formal analysis; methodology; project administration; supervision; validation; visualization; writing—original draft; writing—review and editing. **Bohee Lee**: Conceptualization; data curation; methodology; validation; writing—original draft; writing—review and editing. **Ilari Kuitunen**: Conceptualization; data curation; formal analysis; methodology; software; validation; visualization; writing—original draft; writing—review and editing.

## CONFLICT OF INTEREST STATEMENT

The authors declare no conflicts of interest.

## Supporting information

Supporting Information S1

## Data Availability

All data included were derived from publicly available documents cited in the references. Extracted data are available upon request to the corresponding author.
