# Supplementary material for: Epicutaneous immunotherapy for food allergy: A systematic review and meta‐analysis
Source: Clin Transl Allergy. 2025 Mar 2;15(3):e70045. doi: 10.1002/clt2.70045 (PMC11872371; doi:10.1002/clt2.70045)

**Supporting information – online supplementary material**

**Search strategy**

**Date of the searches:** April 30 and May 20, 2024.

**Databases:** PubMed, Scopus, and EMBASE.

**Search phrase generally:** ((immunotherapy or desensitisation) AND (epicutaneous)) AND (allergy or sensitization)

**PubMed (n = 481):** ((immunotherapy or desensitisation) AND (epicutaneous)) AND (allergy or sensitization) ("immunotherapy"[MeSH Terms] OR "immunotherapy"[All Fields] OR "immunotherapies"[All Fields] OR "immunotherapy s"[All Fields] OR ("desensitise"[All Fields] OR "desensitised"[All Fields] OR "desensitiser"[All Fields] OR "desensitises"[All Fields] OR "desensitising"[All Fields] OR "desensitisation, immunologic"[MeSH Terms] OR ("desensitisation"[All Fields] AND "immunologic"[All Fields]) OR "immunologic desensitisation"[All Fields] OR "desensitisation"[All Fields] OR "desensitisation"[All Fields] OR "desensitisations"[All Fields] OR "desensitize"[All Fields] OR "desensitized"[All Fields] OR "desensitizer"[All Fields] OR "desensitizers"[All Fields] OR "desensitizes"[All Fields] OR "desensitizing"[All Fields])) AND ("epicutaneous"[All Fields] OR "epicutaneously"[All Fields]) AND ("allergie"[All Fields] OR "hypersensitivity"[MeSH Terms] OR "hypersensitivity"[All Fields] OR "allergies"[All Fields] OR "allergy"[All Fields] OR "allergy and immunology"[MeSH Terms] OR ("allergy"[All Fields] AND "immunology"[All Fields]) OR "allergy and immunology"[All Fields] OR ("sensitisation"[All Fields] OR "sensitisations"[All Fields] OR "sensitise"[All Fields] OR "sensitised"[All Fields] OR "sensitiser"[All Fields] OR "sensitisers"[All Fields] OR "sensitises"[All Fields] OR "sensitising"[All Fields] OR "sensitization"[All Fields] OR "sensitizations"[All Fields] OR "sensitize"[All Fields] OR "sensitized"[All Fields] OR "sensitizer"[All Fields] OR "sensitizers"[All Fields] OR "sensitizes"[All Fields] OR "sensitizing"[All Fields]))

**Scopus (n = 414):** TITLE-ABS-KEY ((immunotherapy or desensitisation) AND (epicutaneous)) AND (allergy or sensitization)

**EMBASE:** Embase Classic+Embase <1947 to 2024 May 20>

| #1 | exp immunotherapy/ or exp desensitisation/ | 366036 |
| --- | --- | --- |
| #2 | (immunotherap* or desensiti?).mp. | 340595 |
| #3 | exp patch test/ | 24710 |
| #4 | epicutaneous*.mp. | 3921 |
| #5 | #3 or #4 | 27591 |
| #6 | #2 and #5 | 816 |
| #7 | exp allergy/ or exp hypersensitivity/ or exp sensitization/ | 882581 |
| #8 | (allerg* or hypersensiti* or sensiti?ation or sensiti?e*).mp. | 1804789 |
| #9 | #7 or #8 | 2185945 |
| #10 | #6 and #9 | 784 |

**Supplementary table S1.** Outcome subjectivity and objectivity rating matrix.

| **Study (Trial)** | **Oral food challenged** | **Treatment associated harms** | **Quality of life** | **Lab values** |
| --- | --- | --- | --- | --- |
|  | **Subjective** | **Both** | **Subjective** | **Objective** |
| **Peanut** |  |  |  |  |
| Davis, 2023^19^ (PEPITES and REALISE) | DBPCFC | yes | no | no |
| DunnGalvin, 2021^28^ (PEPITES and PEOPLE) | no | no | yes | no |
| Fleischer, 2019^27^ (PEPITES) | DBPCFC | yes | no | yes |
| Fleischer, 2020^17^ (PEOPLE) | DBPCFC | yes | no | yes |
| Greenhawt, 2023^22^ (EPITOPE) | DBPCFC | yes | no | yes |
| Jones, 2017^29^ (Consortium Food Allergy Research group) | DBPCFC | yes | no | yes |
| Pongracic, 2022^23^ | no | yes | no | yes |
| Sampson, 2017^30^ | DBPCFC | yes | no | yes |
| Scurlock, 2021^18^ (Consortium Food Allergy Research group) | DBPCFC | yes | no | yes |
| **Cow’s milk** |  |  |  |  |
| Dupont, 2010^21^ | OFC | yes | no | yes |
| Petroni, 2024^20^ | DBPCFC | yes | yes | yes |

DBPCFC, double-blind placebo-controlled food challenge; OFC, oral food challenge (not specified otherwise)

**Supplementary figures**

**Supplementary figure 1.** Total treatment-emergent adverse events in peanut EPIT trials.


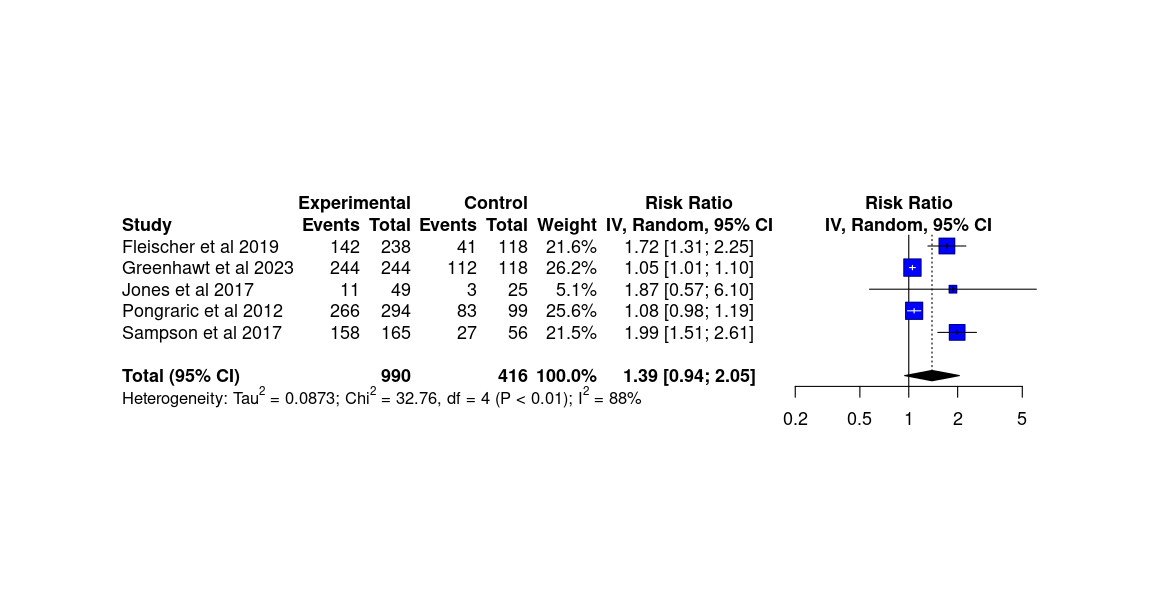


**Supplementary figure 2.** Funnel plot of the possible publication bias in the total treatment-emergent adverse event analysis of peanut studies. Egger’s test p=0.08 indication no clear sign of publication bias.
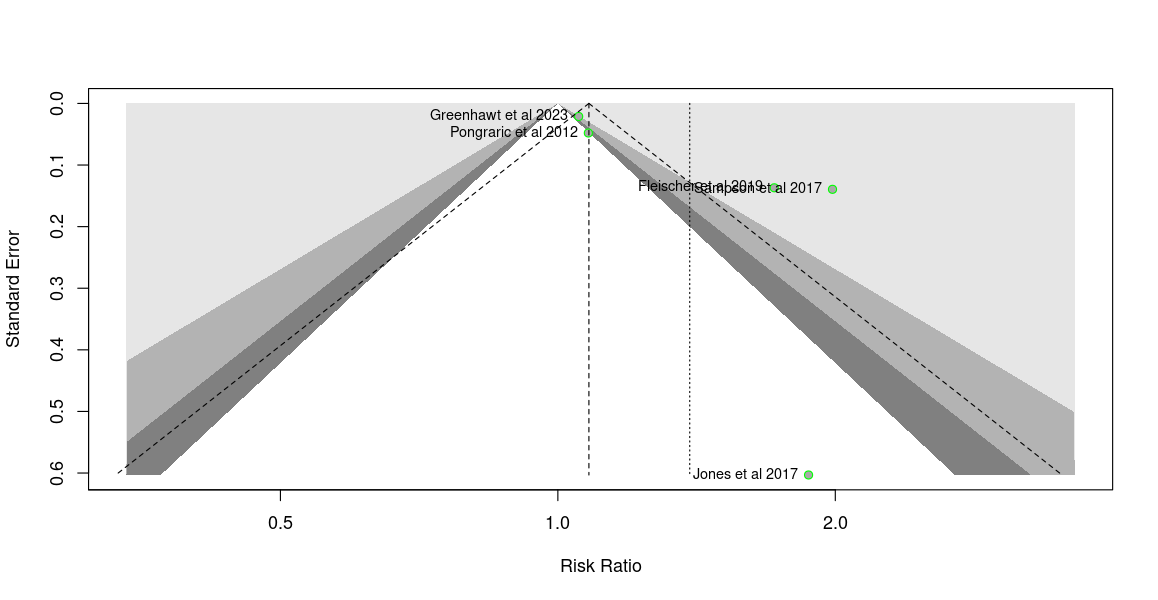


**Supplementary figure 3.** Treatment-emergent mild adverse events in peanut EPIT trials.

**
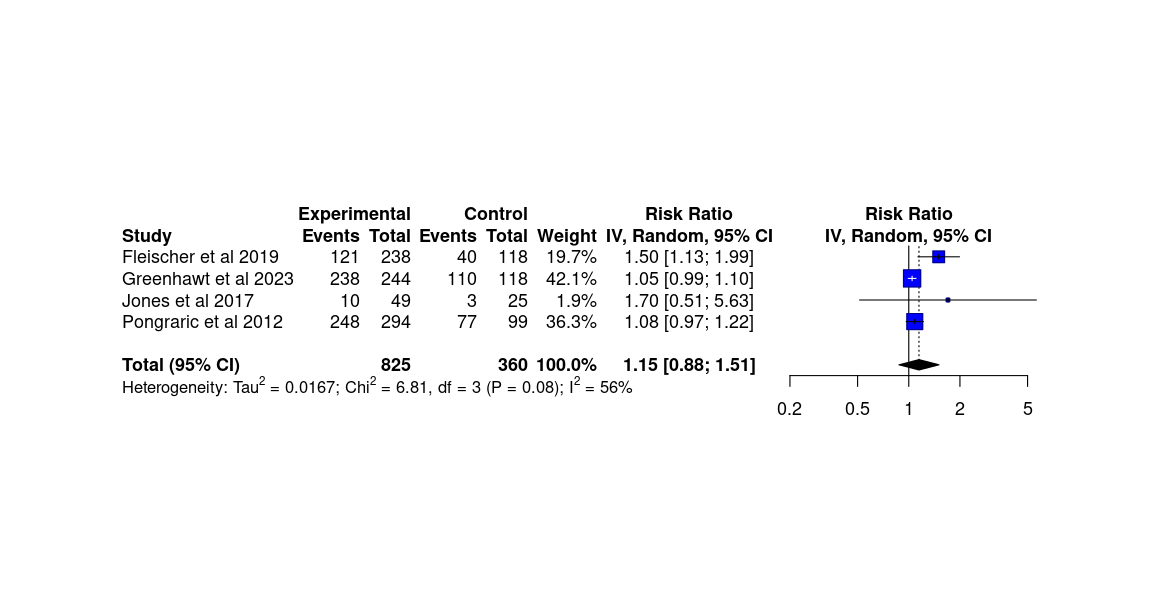
**

**Supplementary figure 4.** Treatment-emergent moderate adverse events in peanut EPIT trials.

**
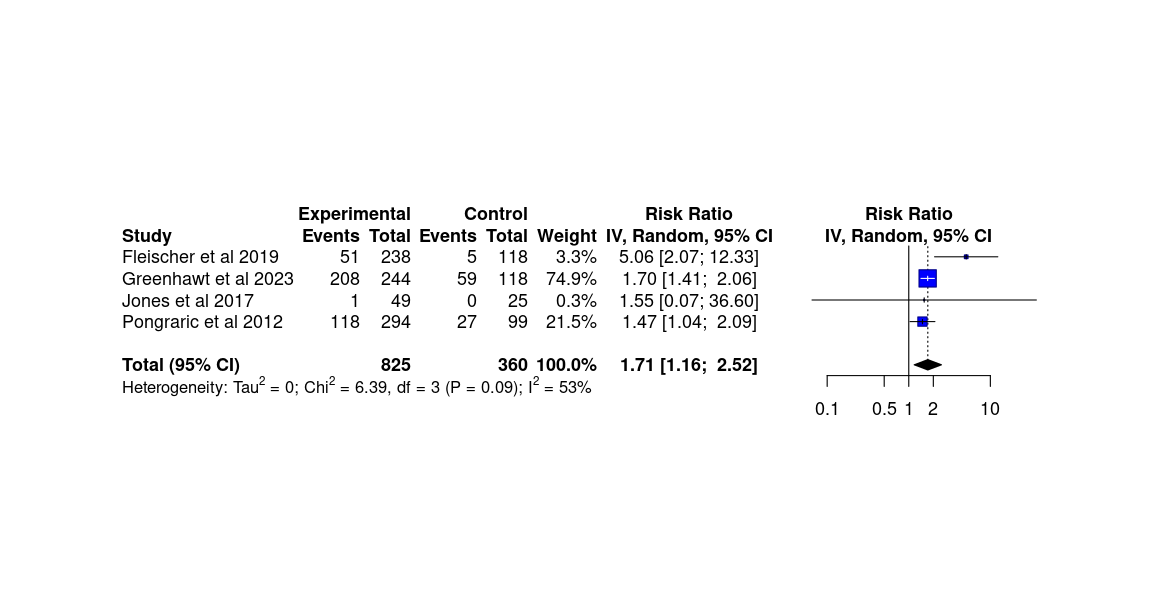
**

**Supplementary figure 5.** Treatment-emergent severe adverse events in peanut EPIT trials.

**
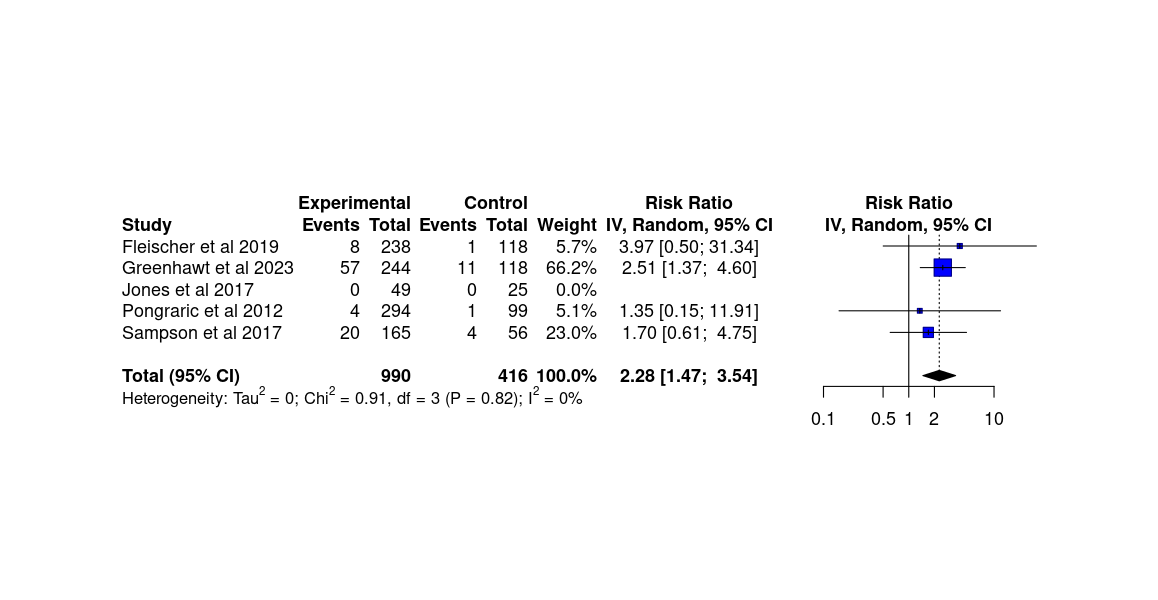
**

**Supplementary figure 6.** Treatment-emergent serious adverse events in peanut EPIT trials.

**
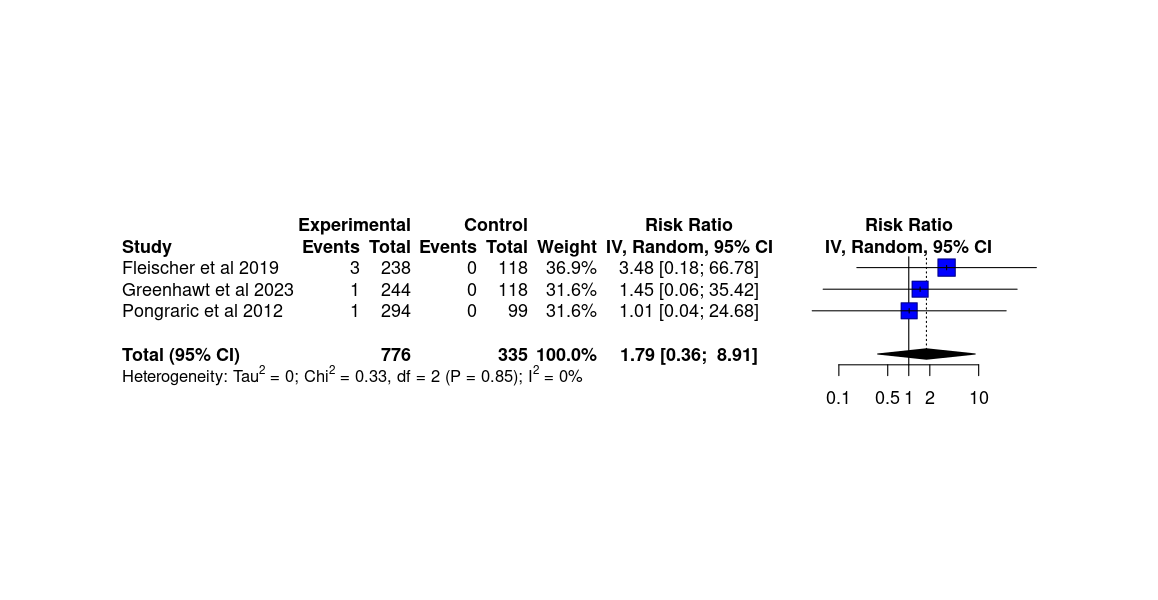
**

**Supplementary figure 7.** Treatment-emergent adverse events of anaphylaxis in peanut EPIT trials.


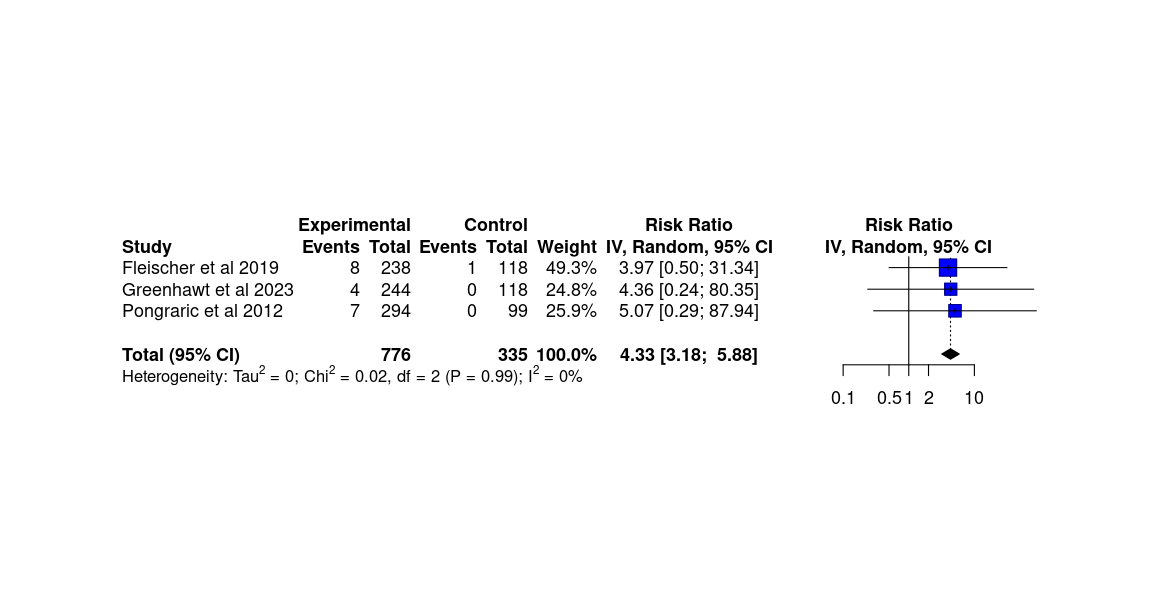


**Supplementary figure 8.** Total treatment-emergent adverse events in cow’s milk EPIT trials.


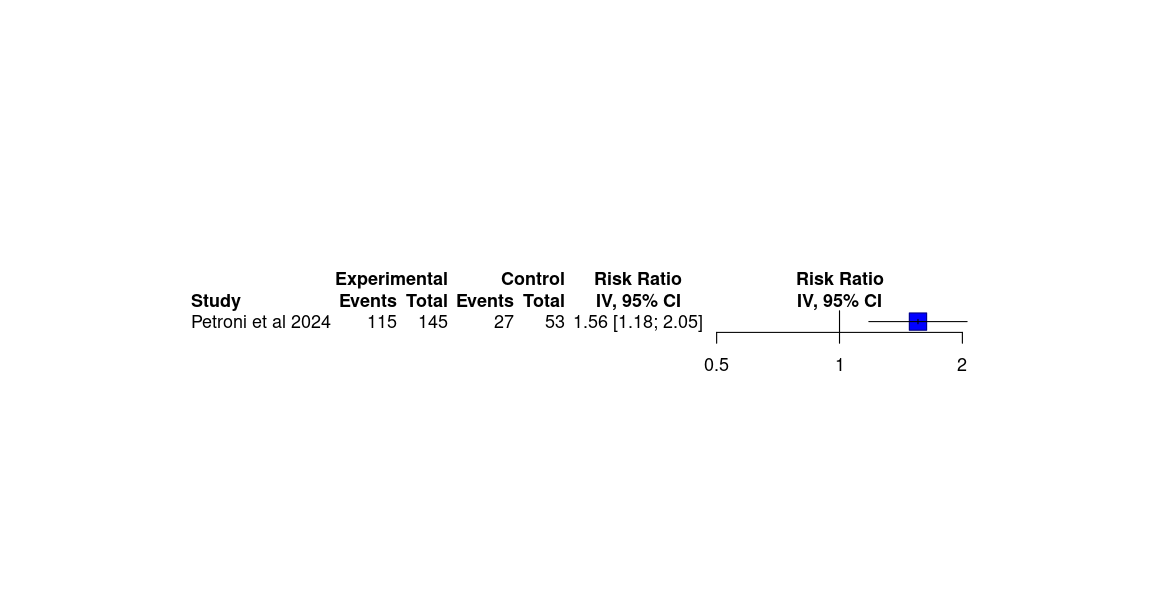


**Supplementary figure 9.** Treatment-emergent adverse events of skin and subcutaneous tissues in cow’s milk EPIT trials.


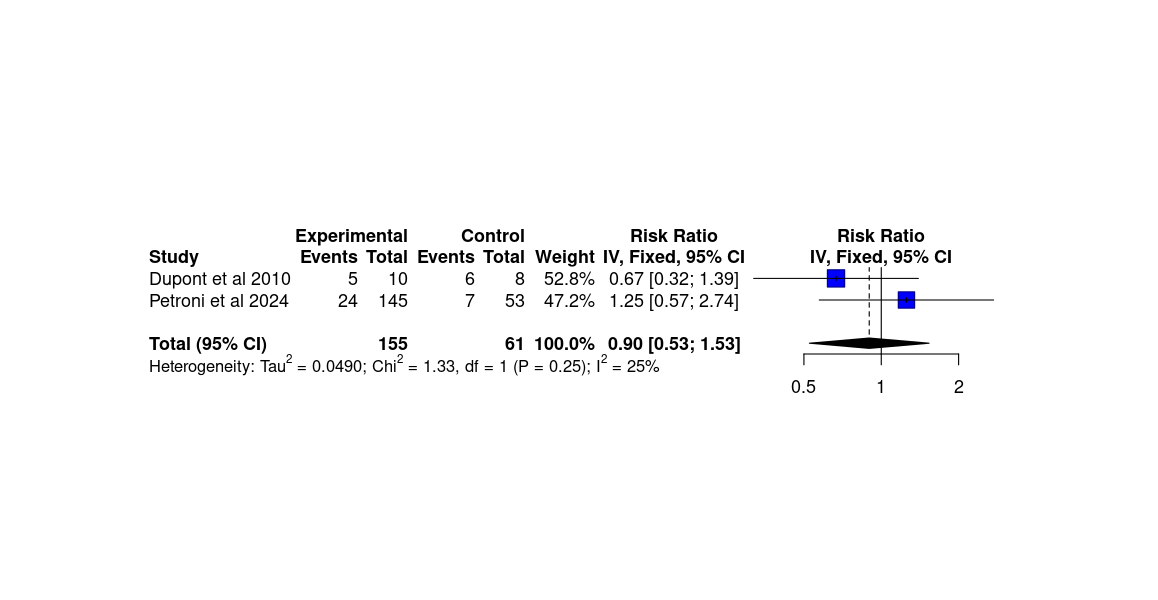


**Supplementary figure 10.** Treatment-emergent adverse events of respiratory tract in cow’s milk EPIT trials.


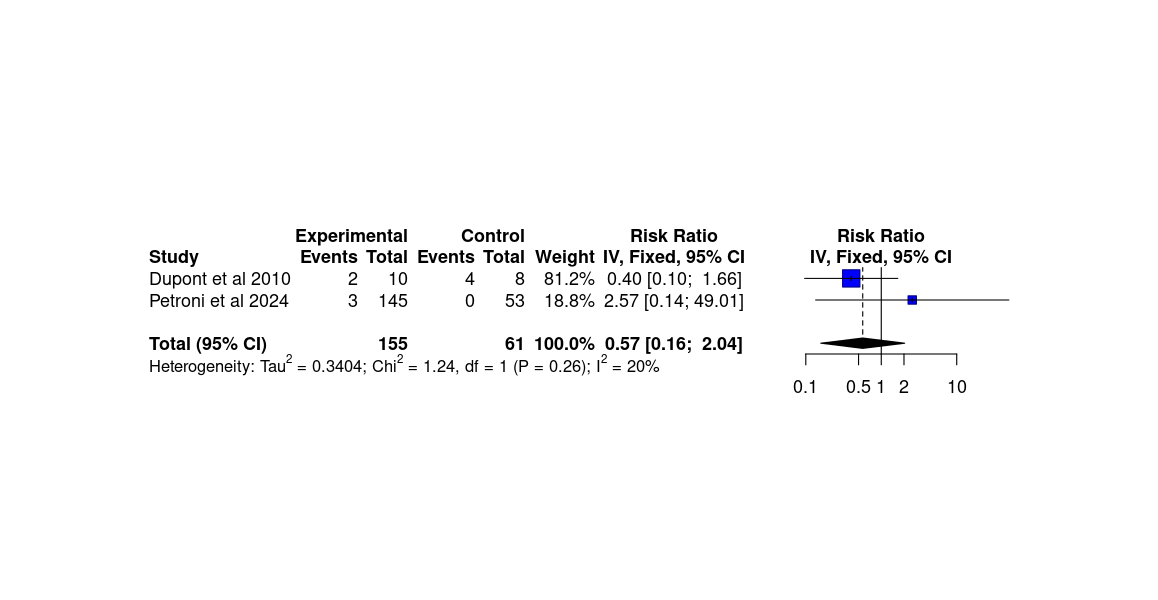


**Supplementary figure 11.** Treatment-emergent adverse events of gastrointestinal tract in cow’s milk EPIT trials.


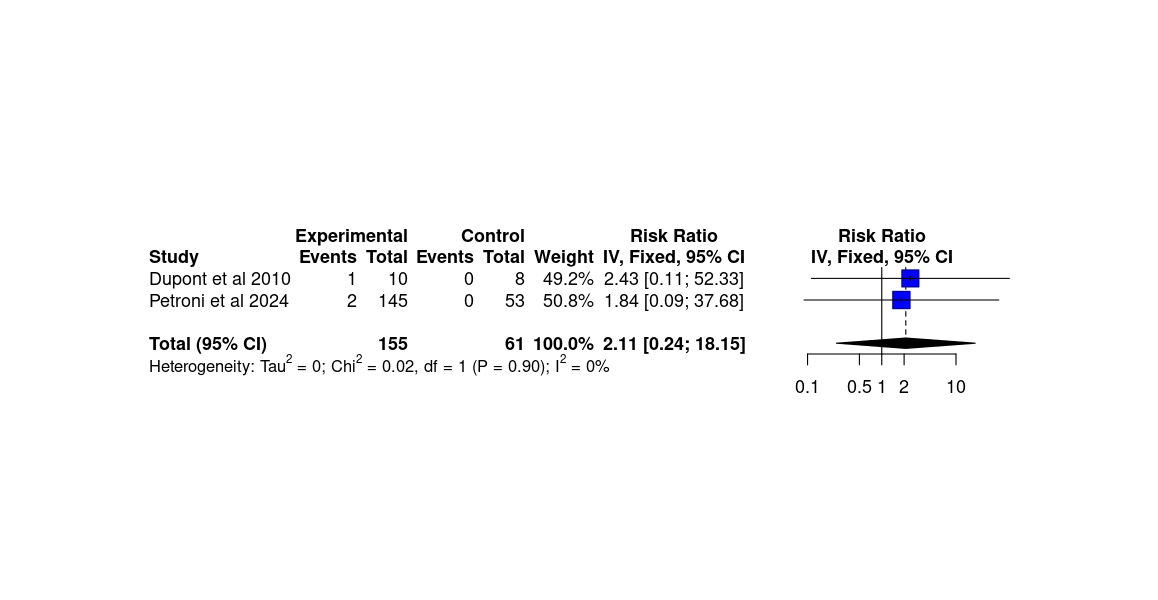


**Supplementary figure 12.** Treatment-emergent adverse events of anaphylaxis in cow’s milk EPIT trials.


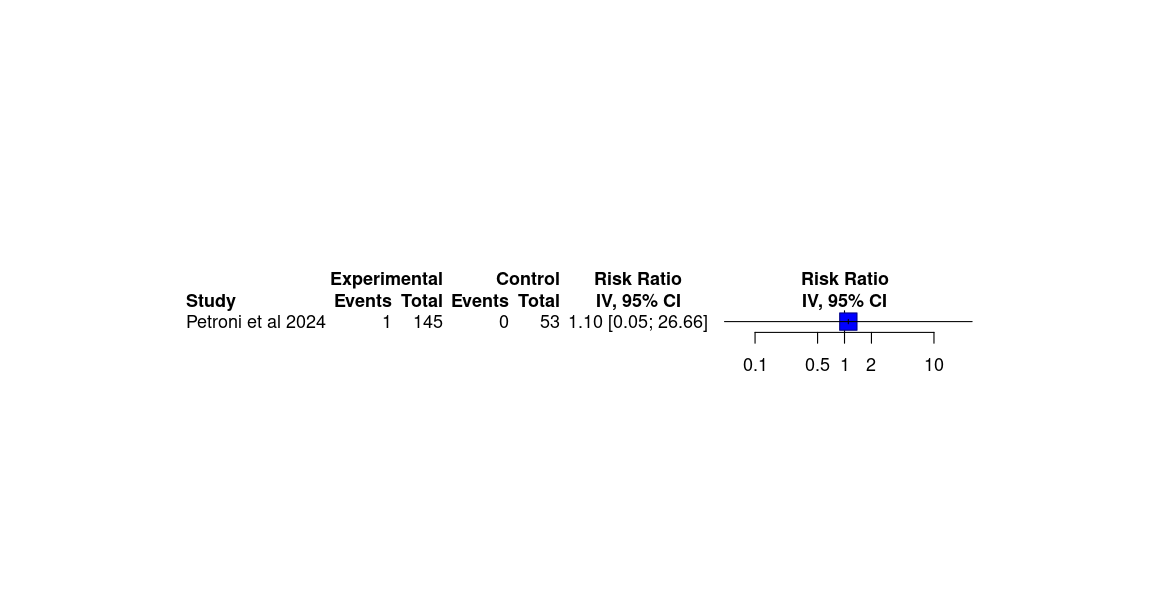

Supplement: Supplementary file 1 — Supporting Information S1 [file CLT2-15-e70045-s001.docx]
